# Prospective evaluation and clinical outcomes of adaptive radiotherapy for locally advanced non-small cell lung cancer (LA-NSCLC)

**DOI:** 10.2340/1651-226X.2026.45745

**Published:** 2026-05-27

**Authors:** Ingvild Helø Carlsen, Vilde Ragnvaldsen Dyvik, Tuva Borgund Johannessen, Kristine Fjellanger, Inger Marie Sandvik, John-Vidar Hjørnevik, Turid Husevåg Sulen, Tone Nybø, Johanna Austrheim Hundvin, Sara Margareta Cecilia Pilskog, Øystein Fløtten, Vilde Drageset Haakensen, Liv Bolstad Hysing

**Affiliations:** a Cancer Clinic, Haukeland University Hospital, Bergen, Norway; b Department of Clinical Science, University of Bergen, Bergen, Norway; c Department of Physics and Technology, University of Bergen, Bergen, Norway; d Department of Thoracic Medicine, Haukeland University Hospital, Bergen, Norway; e Cancer Clinic, Oslo University Hospital, Oslo, Norway

**Keywords:** image-guided radiotherapy, non-small cell lung carcinoma, clinical protocol, computer-assisted radiotherapy planning, survival, locoregional neoplasm recurrence, neoplasm metastasis, adverse effects

## Abstract

**Background and purpose:**

Locoregional tumor control is strongly associated with survival for non-small cell lung cancer, emphasizing the importance of precise radiotherapy delivery. This study aims to evaluate a traffic light adaptation protocol regarding target coverage, protocol performance and patient outcomes.

**Material and methods:**

From 2019 to 2022, we prospectively enrolled inoperable non-small cell lung cancer patients. We acquired daily cone-beam computed tomography and traffic light registrations, classifying anatomical changes and need of action. Using repeated computed tomography (rCT) in week 1 and 3 for dosimetric evaluation, we recalculated clinical target volume (CTV) D98% and V95% with and without adaptation, and compared traffic light registrations with the actual dose distribution. Protocol performance was evaluated by the sensitivity and specificity in detecting CTV V95% < 98%.

**Results:**

Among 45 patients with complete registrations, baseline shift and tumor shrinkage were most common changes, leading to corrections from bone to tumor match in 37.8% and replanning in 13.3% of patients. Among 38 patients with at least one rCT, 15.8% would have received insufficient CTV coverage without adaptation. Protocol sensitivity, specificity and balanced accuracy were 83.3, 88.6, and 85.7%, respectively. For 38 stage II–III patients, median overall survival was 43.5 months (95% confidence interval [CI]: 32.9–54.1), median time to locoregional failure 27.4 months (95% CI: 0–61.8) and median estimated time to distant failure 30.7 months (CI not estimated due to censoring).

**Interpretation:**

The protocol identified relevant changes, improving target coverage without increasing organ at risk doses. With appropriate training, protocol performance was good, and clinical outcomes were consistent with international results.

## Introduction

For patients with inoperable locally advanced non-small cell lung cancer (LA-NSCLC), standard of care is concurrent chemoradiotherapy (CRT), followed by consolidation immunotherapy (ICI) for a subset of patients [1, 2]. Locoregional control after treatment is strongly associated with progression-free and overall survival (OS) [3–5], underscoring the importance of accurate radiotherapy delivery. The radiation part of the treatment, however, presents challenges due to respiratory motion and anatomical changes throughout the treatment course, but adaptive radiotherapy (ART) has potential to improve precision and enhance outcomes [6–9].

Daily monitoring of anatomical changes using onboard cone-beam computed tomography (CBCT) for offline adaptation remains the most common strategy to perform ART [10]. However, only a limited number of studies have evaluated the use of traffic light protocols as selection tools for identifying patients who require adaptation in clinical practice [8, 11–13], and traffic light protocols with extensive criteria designed to capture all potential anatomical deviations may result in high false positive (FP) rates, thereby increasing the workload of medical physicists and oncologists [12].

In 2019, we initiated a prospective study for LA-NSCLC patients where we implemented a traffic light-based adaptive protocol and acquired computed tomography (CT) images during treatment to evaluate protocol performance. With 3 years of follow-up, our objective is to present the protocol’s impact on delivered clinical target volume (CTV) dose, its ability to detect anatomical changes relevant for CTV dose coverage, and clinical outcomes.

## Material and methods

### Study participants

From 2019 to 2022, we invited inoperable NSCLC patients referred to CRT at Haukeland University Hospital to participate in this prospective observational study. The study was approved by the Regional Committee for Medical and Health Research Ethics (protocol code 2019/749), and all participants provided informed consent.

### Treatment and study design

All patients had prescribed doses of 60–70 Gy in 2 Gy fractions. We delivered the treatment with intensity-modulated radiotherapy (IMRT) or volumetric modulated arc therapy (VMAT) in either free breathing (FB) as standard or deep inspiration breath hold (DIBH) as optional. The planning procedure has been described in detail [14, 15]. Briefly, we planned patients treated in FB based on a 10-phase four-dimensional computed tomography (4DCT), with internal gross tumor volume (IGTV) including gross tumor volume (GTV) positions on all phases, and planned patients treated with DIBH based on three repeated DIBH scans, with IGTV including GTV positions on all three scans. We defined the CTV by expanding the IGTV by 5-mm margins, without extending into uninvolved organs, and the planning target volume (PTV) by expanding the CTV by 5-mm isotropic margins. Delineations were performed by the responsible oncologist according to European Society for Radiotherapy and Oncology (ESTRO) guidelines [16], and organ at risk (OAR) volumes were delineated according to Radiation Therapy Oncology Group (RTOG) guidelines [17]. We delivered DIBH treatment using the Varian respiratory gating system with Visual Coaching Device (Varian Medical Systems, Siemens), applying a 2–3 mm gating window.

Daily image guidance was performed using CBCT with bone match as default. For FB treatments, we acquired CBCTs during normal respiration, whereas for DIBH treatments, we obtained each CBCT over 2–3 breath-holds of approximately 20 seconds each. We used our CBCT-based traffic light protocol (detailed in Supplementary material) to detect anatomical changes and guide treatment adaptations. Interpretation of CBCT images was performed by the radiation therapy technologist (RTT) within 15 minutes of each treatment session. We developed the protocol based on the work of Kwint et al. [18] and Møller et al. [19], incorporated local adjustments, and implemented it specifically for this study. Implementation was done without prior RTT training. After treating the first 19 patients, consistency in interpretation was re-evaluated by offline review [20]. All 15 RTTs involved in the treatment were invited to participate in an offline re-evaluation of the two fractions that were followed by a control-CT image the same day. The results from this re-evaluation were reviewed during an internal teaching session, and relevant cases were discussed. For the remaining patients, the clinical online registrations were relied upon.

For the traffic-light protocol (detailed in Supplementary), the RTTs recorded six predefined anatomical changes at every fraction; atelectasis, pleural effusion, infiltrative changes, baseline shift, tumor growth or tumor shrinkage, and assigned the findings a traffic light color to indicate a required need of action. Large changes (e.g., tumor masses likely outside PTV) were assigned red, and treatment was paused until reviewed. Clear anatomical changes (e.g., atelectasis or tumor growth near the PTV border) were assigned orange. The fraction was delivered but required a physicist visual review of dose distribution (control CBCT task), followed by the oncologist’s decision on adaptation. Smaller anatomical changes (e.g., baseline shift of 2–5 mm or tumor shrinkage 1–3 cm) were assigned yellow with no need for action, while even smaller or no anatomical changes were assigned green also with no need for action. In this study ART was defined as (1) corrections from default bone match to tumor match, (2) replanning, and (3) breathing instructions tailored to the patient with focus on reproducing the breathing pattern/DIBH at planning.

To evaluate protocol performance, we quantified the frequency of color registrations, type of anatomical changes, number of control CBCT tasks initiated and types of adaptations. We used a cross-sectional study design with a repeated computed tomography (rCT) during week 1 and another during week 3. At these two time points, one experienced oncologist re-delineated all target volumes for all patients. To estimate relevant dose parameters for the patient in question, we recalculated dose on the rCTs using corresponding replans and fraction specific iso-center shifts for ART, and the original plan with bone match for no-ART. We quantified ART performance as improvement in CTV dose coverage (D98% and V95%).

We estimated the sensitivity, specificity and accuracy of the traffic light protocol in detecting CTV miss, defined as V95% < 98% on the rCTs. For this analysis, we used the per patient traffic light registrations performed on the corresponding same day CBCTs. True positive (TP) was defined as CTV V95% < 98% with corresponding red or orange traffic lights. FP was defined as CTV V95% > 98% with corresponding red or orange traffic lights. True negative (TN) was defined as CTV V95% > 98% with corresponding yellow or green traffic lights. False negative (FN) was defined as CTV V95% < 98% with corresponding yellow or green traffic lights.

Clinical follow-up was according to national guidelines [21]. Index date for follow-up was set to radiation start. We retrieved dates for locoregional failure (LRF), distant failure (DF), and death, as well as information on radiation-related toxicities during treatment, at 3 months, 6 months, and 3 years. We graded radiation-related toxicity according to the Common Terminology Criteria for Adverse Events version 5 (CTCAE v5.0). LRF was defined as regrowth of the irradiated tumor or lymph nodes or the appearance of new malignancy-suspected lesions within the 50% isodose [22], while new lesions in the contralateral lung were defined as distant metastasis. End of observation was December 1, 2025. For the OS analysis, patients still alive at this date were censored. For the LRF and DF analyses, patients without LRF or DF were censored either at death or December 1, 2025.

### Statistical analysis

We summarized data using descriptive statistics including frequencies, medians, and interquartile range (IQR). We compared dose distributions between ART and no-ART using the Wilcoxon signed-rank test for related samples in SPSS version 30.0. We used R version 4.5.0 to estimate median OS, time to LRF and DF with Kaplan–Meier methods, stratified by ICI-status. Comparison between the ICI and no-ICI group was done using the log-rank test. Significance was defined as *p*-value < 0.05.

## Results

### Study participants and treatment compliance

**Figure 1:**
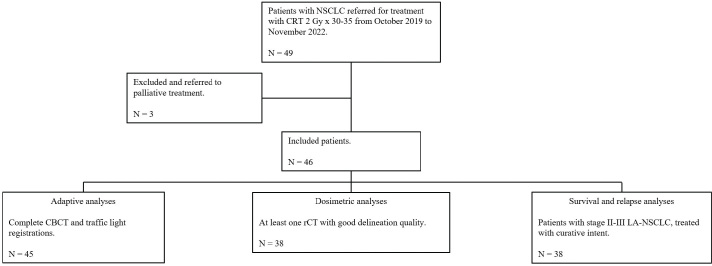
Consort diagram of patients included in the study. NSCLC: non-small cell lung cancer; CRT: chemoradiotherapy; CBCT: cone-beam computed tomography; rCT: repeated computed tomography.

A total of 49 patients were enrolled from 2019 to 2022, and 45, 38, and 38 had data eligible for adaptive, dosimetric, and survival analyses, respectively (Figure 1).

Patient and treatment characteristics for all 46 patients receiving treatment (Table 1) show that most patients had stage II–III disease (89.1%). Most patients (89.1%) received concurrent treatment, while a smaller proportion (10.9%) received sequential treatment. Most patients (71.7%) were treated in FB, while a smaller proportion (28.9%) were treated in DIBH due to large respiratory motion or exceeded lung dose constraints in FB. Treatment compliance was good with most patients (95.7%) receiving 60–70 Gy. Two patients stopped treatment early, one died due to sepsis caused by Pseudomonas aeruginosa, and the other was hospitalized and discontinued treatment due to severe radiation esophagitis and sepsis of unknown pathogen.

**Table 1:** Patient and treatment characteristics for all 46 patients receiving treatment.

Patient characteristics *n* = 46	Value
Gender, *n* (%)	
Male	26 (56.5%)
Female	20 (43.5%)
Age, years	
Median (IQR)	65.5 (61.3–70.8)
Performance status, *n* (%)	
0	22 (47.8%)
1	24 (52.2%)
2	0
Smoking habits, *n* (%)	
Non-smoker	0
Smoker	17 (37.0%)
Previous smoker	29 (63.0%)
*Stage, *n* (%)	
IA	1 (2.2%)
IB	1 (2.2%)
IIA	1 (2.2%)
IIB	2 (4.3%)
IIIA	19 (41.3%)
IIIB	17 (37.0%)
IIIC	2 (4.3%)
IV	3 (6.5%)
Histology, *n* (%)	
Adenocarcinoma	16 (34.8%)
Squamous cell carcinoma	22 (47.8%)
Large cell neuroendocrine carcinoma	1 (2.2%)
Other and unclassified carcinoma	7 (15.2%)
**PD-L1 expression, *n* (%)	
≥ 1%	31 (67.4%)
< 1%	14 (30.4%)
Cardiac comorbidity, *n* (%)	
No cardiac comorbidity	38 (82.6%)
Cardiac comorbidity	8 (17.4%)
Pulmonary disease, *n* (%)	
No pulmonary disease	19 (41.3%)
Pulmonary disease	27 (58.7%)
FEV1	
Median L (IQR)	2.07 (1.81–2.46)
Median % of predicted value (IQR)	71.5 (64.3–87.8)
DLCO	
Median % of predicted value (IQR)	65.0 (55.3–76.5)
Tumor size, cm	
Median (IQR)	5.05 (3.3–6.88)
Tumor location, *n* (%)	
Left upper lobe	8 (17.4%)
Left lower lobe	11 (23.9%)
Right upper lobe	16 (34.8%)
Right middle lobe	0
Right lower lobe	8 (17.4%)
Right upper and middle lobe	1 (2.2%)
Right lower and middle lobe	1 (2.2%)
No primary tumor	1 (2.2%)
Chemotherapy, *n* (%)	
Concurrent	41 (89.1%)
Sequential	5 (10.9%)
***Immunotherapy, *n* (%)	
No immunotherapy	17 (37.0%)
Consolidation immunotherapy	29 (63.0%)
Radiotherapy prescription, *n* (%)	
60 Gy	16 (34.8%)
66 Gy	29 (63.0%)
70 Gy	1 (2.2%)
Image-guided radiotherapy (IGRT) technique, *n* (%)	
FB	33 (71.7%)
DIBH	13 (28.3%)
****Gross tumor volume primary tumor (GTVp), mL	
Median (IQR)	42.2 (16.6–96.5)
*****Gross tumor volume lymph nodes (GTVn), mL	
Median (IQR)	10.4 (4.8–21.4)
Clinical target volume (CTV), mL	
Median (IQR)	183 (105.6–258.1)
Planning target volume (PTV), mL	
Median (IQR)	324.6 (212.7–436.8)
Planned OAR doses, Gy	
Mean heart dose, median (IQR)	10.03 (5.22–13.58)
Mean lung dose, median (IQR)	14.23 (12.72–16.64)
Mean esophageal dose, median (IQR)	20.34 (15.58–25.67)
Overall treatment time, days	
Median (IQR)	44 (41–44)

* Two patients had single brain metastases, treated with surgery and Gamma Knife prior to inclusion, and one had a single bone metastasis in the scapula which received fractionated radiotherapy in curative doses.

** One patient was not tested for PD-L1 due to insufficient histological material.

*** According to national guidelines, adjuvant durvalumab is only approved for patients with PD-L1 ≥ 1%. One of the PD-L1 positive patients had received concurrent pembrolizumab and did therefore not received adjuvant durvalumab.

**** Two patients did not have GTVp delineated on the planning CT.

***** One patient did not have GTVn delineated on the planning CT. IQR: interquartile range; FB: free breathing; DIBH: deep inspiration breath hold; OAR: organ at risk; PD-L1: Programmed Death-Ligand 1; FEV1: Forced Expiratory Volume in 1 second; DLCO: Diffusing Capacity of the Lung for Carbon Monoxide.

### Prospective evaluation of adaptive protocol

A total number of 1413 traffic light classifications were registered clinically. Of these, 446 (31.6%) were green, 719 (50.9%) were yellow, 248 (17.6%) were orange, and 0 (0%) was red. Out of 30–35 fractions per treatment course, green color was assigned to a median of 8 fractions (IQR 3–15), yellow to a median of 18 fractions (IQR 9–22), orange to a median of 3 fractions (IQR 0–7), and red to 0 fractions. Inconsistency was found in interpretation when re-evaluating the first 19 patients. Twelve out of 15 RTTs involved in patient treatment participated in the re-evaluation, and a median of ten patients (range 1–19) was evaluated per RTT. After re-evaluation, 24 fractions were labeled the same color, 8 changed from green to yellow, 1 from green to orange, 1 from green to red, 1 from yellow to orange, and 2 from orange to yellow. The updated colors were used in further analysis to correct for bias in interpretation.

Baseline shift and tumor shrinkage were the most common anatomical changes reported in 93.3 and 40.0% of patients, respectively, although not necessarily to an extent triggering a control CBCT task or an intervention. At least one control CBCT task was initiated in 75.6% of the patients, and 22.2% had more than two tasks initiated.

Adaptations were initiated in 46.7% of the patients; 37.8% were changed from bone to tumor match, 6.7% received breathing instructions, and 13.3% were replanned (Table 2). Thirteen patients were adapted only once, while eight patients needed two or more adaptations. Regarding the six patients who were replanned, only one needed two replans, and the second replan was because the patient changed from DIBH treatment to FB treatment.

**Table 2:** Overview of reported anatomical changes and adaptations among the 45 patients with complete CBCT and traffic light registrations.

Anatomical changes and adaptation	Value
Type of anatomical change, *n* (%)	
Baseline shift	42 (93.3%)
Atelectasis	8 (17.8%)
Tumor growth	3 (6.7%)
Tumor shrinkage	18 (40.0%)
Pleural effusion	0
Infiltrative changes	0
Type of adaptation, *n* (%)	
Adjustment to tumor match	17 (37.8%)
Breathing instructions	3 (6.7%)
Replan	6 (13.3%)

Note that the same patient could have multiple types of changes and types of adaptations. CBCT: cone-beam computed tomography.

**Figure 2:**
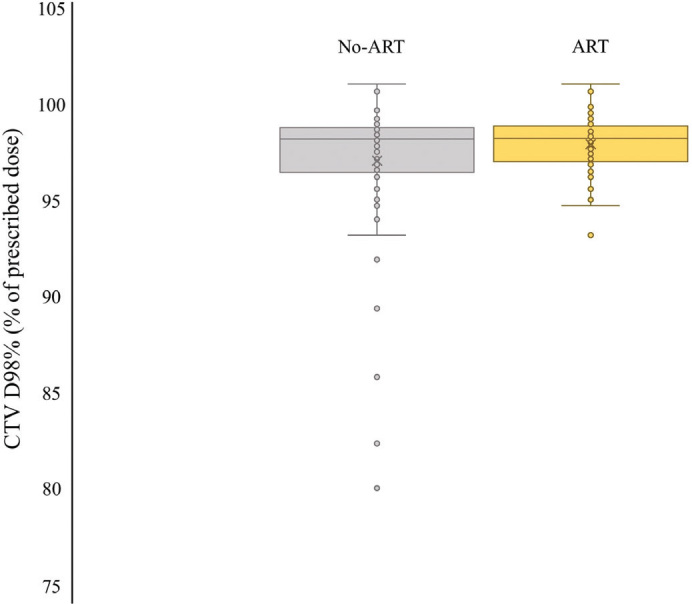
Adaptation analysis of the 38 patients with at least one rCT. CTV D98% was significantly lower without ART compared to ART (Wilcoxon signed-rank test for related samples, *p* = 0.031). rCT: repeated computed tomography; CTV: clinical target volume; ART: adaptive radiotherapy.

The dosimetric analyses showed that the use of ART significantly improved CTV D98% compared to no-ART (Wilcoxon signed-rank test for related samples, standardized test statistic *Z* = 2159, *p* = 0.031) (Figure 2).

**Figure 3:**
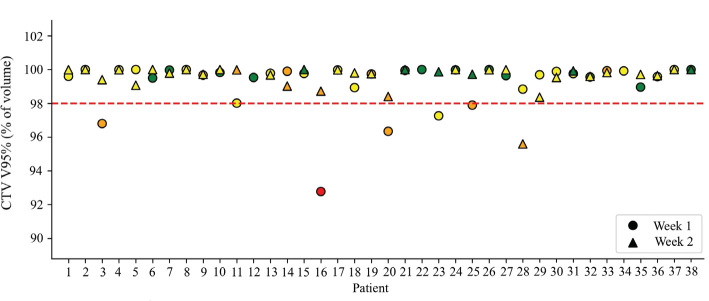
Target coverage in terms of CTV V95% in the recalculated plans from the rCT scans in week 1 and week 3 for the 38 patients with at least one rCT. The color of each time point shows the traffic light color assigned to the treatment fraction using registrations from one experienced RTT for patient 1–19 and registrations by multiple RTTs for patient 20–38. rCT: repeated computed tomography; CTV: clinical target volume; RTT: radiation therapy technologist.

Without ART, six patients (15.8%) would have target miss at one time point (Figure 3). In total, 13 evaluated fractions were assigned orange or red, of which 5 had poor target coverage (TP) and 8 had sufficient coverage (FP). Fifty-nine evaluated fractions were correctly assigned green or yellow (TN) and 1 evaluated fraction was incorrectly assigned yellow (FN). The sensitivity and specificity of the traffic light protocol were 83.3 and 88.6%, respectively, giving a balanced accuracy of 85.7%.

For the OARs, mean doses to the esophagus (MED), lungs (MLD), and heart (MHD) were similar with and without ART (*Z* = 0.014, *p* = 0.989; *Z* = –0.404, *p* = 0.686; *Z* = 0.014, *p* = 0.989, respectively), while the maximum dose to the spinal canal (D0.01cc) was slightly lower with ART (*Z* = –2.206, *p* = 0.027).

### Clinical follow-up

**Figure 4:**
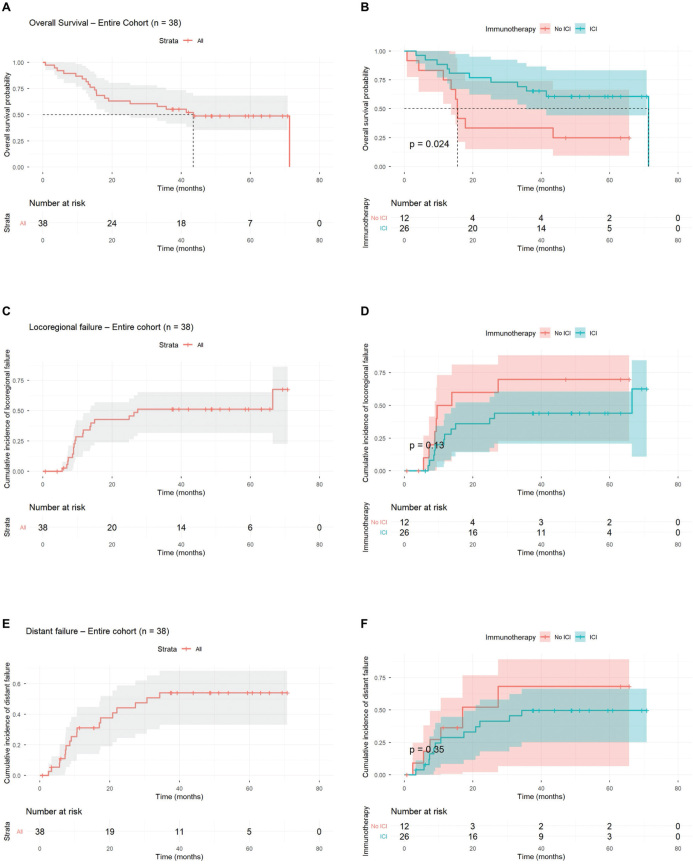
Clinical outcomes for 38 patients with LA-NSCLC disease stage II–III. (A) Median OS of 43.5 months (95% CI: 32.9–54.1) for the entire cohort. (B) Median OS of 71.3 months (95% CI not estimated due to censoring) and 15.4 months (95% CI: 14.4–16.5) in the ICI and no-ICI group, respectively. (C) Median time to LRF of 27.4 months (95% CI: 0–61.8) for the entire cohort. (D) Median time to LRF of 66.5 months (95% CI: 0–136.6) and 9.5 months (95% CI: 2.5–16.5) in the ICI and no-ICI group, respectively. (E) Median estimated time to DF of 30.7 months (95% CI not estimated due to censoring) for the entire cohort. (F) Median estimated time to DF not reached and 17.0 months (95% CI: 0–35.2) in the ICI and no-ICI group, respectively. LA-NSCLC: locally advanced non-small cell lung cancer; CI: confidence interval; ICI: consolidation immunotherapy; LRF: locoregional failure; DF: distant failure.

Median OS was 43.5 months (95% confidence interval [CI]: 32.9–54.1) for the 38 stage II–III patients. The Programmed Death-Ligand 1 (PD-L1) negative no-ICI group had significantly shorter OS (Figure 4). Median time to LRF was 27.4 months (95% CI: 0–61.8) for the entire cohort, and median estimated time to DF was 30.7 months (95% CI not estimated due to censoring) for the entire cohort.

During treatment, 16 (34.8%) and seven (15.2%) patients experienced grade 2 and 3 esophagitis, respectively. One patient (2.2%) developed grade 2 pneumonitis. At 6 months of follow-up, six (13.0%) and three (6.5%) patients had grade 2 and 3 pneumonitis, respectively, while none had grade 2 or 3 esophagitis. There were no grade 4 or 5 toxicities throughout the whole follow-up period (detailed in Supplementary).

## Discussion and conclusion

This prospective observational study demonstrates that our traffic light-based adaptive protocol effectively identifies clinically relevant anatomical changes in patients with LA-NSCLC, improving target coverage without increasing OAR doses. The study shows that for most patients, the original treatment plan is robust. However, for the 13.3% of patients who required replanning, adaptive interventions appear to be clinically relevant.

During implementation, we observed a need for initial observer training to achieve consistent interpretation and reporting of daily CBCT findings. This was accounted for in the analysis, by using updated traffic light colors, and the protocol performance should be interpreted in this context. With appropriate training, we believe that daily CBCTs and use of the traffic light protocol as decision support, are sufficient to enable reliable anatomical adaptation.

In our study, we applied a relatively broad definition of ART, encompassing not only plan adaptation, but also positional adjustments from bone match to tumor match and breathing instructions. If we restrict the definition to replanning alone, 13.3% of our patients required a new treatment plan. These findings are consistent with previous studies, where Møller et al. [19], Bjaanæs et al. [23], and Hattu et al. [12] found adaptive replanning indicated in 12, 18, and 14% of patients, respectively. In these studies, treatment was delivered in FB, and neither breathing instructions nor changes in setup matching were defined as part of ART. We observed that meaningful improvements could be achieved through matching adjustments rather than more resource-intensive replanning, but in cases of baseline shift > 5 mm, matching on tumor rather than bony anatomy required careful balancing between tumor target coverage and the risk of lymph node target miss and spinal canal overdose.

Møller et al. and Bjaanæs et al. used checklists with predefined criteria to guide decisions on adaptation, while Hattu et al. used a traffic light protocol. Direct comparison between the use of checklists and traffic light protocols is challenging due to different criteria and triggering thresholds for adaptation. Hattu et al. optimized their traffic light protocol by omitting criteria triggering control CBCT tasks but not resulting in replanning. This resulted in a 23% reduction in CBCT control tasks without affecting the proportion of patients who underwent replanning. In our study, protocol performance was high, with the same proportions of patients undergoing replanning, indicating a well-functioning protocol.

The traffic light protocol implemented in this study represents formalization and structuring of previously existing clinical assessment procedures at our institution. Based on the study design, our findings indicate that this formalized protocol is feasible and well-functioning in clinical practice. The study was mainly designed to compare ART vs no-ART, not to compare the use of traffic lights vs no traffic lights. We therefore cannot exclude that similar CBCT-based changes could have been identified without a formalized traffic light protocol. To our knowledge, there are currently no studies directly comparing the use of traffic light protocols to unformalized assessment of CBCT-based changes. While other approaches may be equally effective, our results support that the proposed traffic light protocol represents one well-functioning way of identifying adaptation needs.

With respect to OAR dosimetry, dose constraints were largely respected irrespective of whether ART was applied or not. With ART, MED was below our constraint of 34 Gy in 92.1% of patients and MLD was below our constraint of 20 Gy in 97.4%. MHD was below 25 Gy in 100% of patients. Three patients with MED > 35 Gy were assigned green, yellow, and orange, while one patient with MLD slightly above 20 Gy was assigned green. Two patients had spinal canal maximum dose (D0.01cc) slightly above our clinical constraint of 50 Gy, and both were assigned orange. Future work may focus on increasing the protocol’s sensitivity in detecting OAR overdose; however, dose constraints were largely met with no significant difference between ART and no-ART, indicating an acceptable safety profile in the present protocol.

Regarding the time points chosen for rCTs in week 1 and 3, one would expect this to primarily reflect positional and volumetric changes, respectively. However, we observed that tumor shrinkage already occurred by week 1. A possible explanation for this is that many patients received their first chemotherapy cycle prior to radiation start, and a smaller proportion of the patients got sequential radiotherapy, which may have resulted in earlier visible tumor response. Zhou et al. identified the dose range of 30–50 Gy (15–25 fractions, week 3–5) to be the most appropriate time window for ART based on tumor regression [10]. However, these patients predominantly received concurrent CRT without induction cycles. Earlier studies attempting to characterize patterns of tumor regression have several methodological limitations, including less advanced imaging techniques, small sample sizes, and heterogeneity in treatment intent and delivered dose [24].

ART based on functional imaging with FDG-PET or diffusion-weighted MRI has been explored for enabling biological response-adapted treatment strategies [25, 26], but the primary objective in standard fractionated photon radiotherapy of NSCLC remains accurate delivery of planned dose. In this context, our results indicate that an offline CBCT-based approach is effective and well suited for clinical workflows. The use of online CBCT-based adaptation has also shown promising results [27], but whether online adaptation provides clinically meaningful advantages over systematic offline monitoring remains to be established.

Several limitations of this study warrant consideration. First, as this was a single-center study, we acknowledge that the relatively small sample size constitutes a limitation. Second, after treating the 19 first patients, inconsistency in CBCT interpretation was addressed with internal teaching sessions, case discussions, and re-evaluation. Even though the clinical online registrations were relied upon for the remaining 26 patients, there was no formal re-evaluation of consistency after the training, and we acknowledge that this represents a limitation of the study. Third, a small sample size with few positive findings (five TP and one FN) makes the sensitivity rather unreliable. To partly address this statistical challenge, we reported a balanced accuracy as a performance metric. Nevertheless, we acknowledge that this affects the validity of our results, which should be validated in a larger cohort. Lastly, we recognize that there can be differences in breathing and positioning between onboard CBCTs and rCTs, which will always entail a certain degree of uncertainty.

Although this study was not designed to assess survival benefits attributable to ART, we compared our findings against real-world stage III patients [28]. In summary, real-world larger multicenter analyses demonstrate considerable variability in survival outcomes, and our results fall within this reported range. The RELEVANCE study assessed 487 inoperable stage III NSCLC patients with index date at start of CRT. Median OS was 44.6 months (95% CI: 36.9—not reached) and 21.3 months (95% CI: 17.5–28.1) in the ICI and no-ICI group, respectively [29]. The SPOTLIGHT study assessed 469 inoperable stage III NSCLC patients with index date at end of CRT. Median OS was not reached and 19.4 months (95% CI: 11.7–24.0) in the ICI and no-ICI group, respectively [30]. An important distinction in treatment exposure is that none of our PD-L1 negative patients received ICI according to national guidelines, whereas PD-L1 negative patients were treated with ICI in both RELEVANCE and SPOTLIGHT. With the recognition of ICI also being beneficial for PD-L1 negative patients [31], this could have contributed to poorer outcomes in our no-ICI group, relatively to the two other studies.

In conclusion, this prospective observational study provides a useful addition to the existing literature and suggests that our traffic light-based adaptive protocol effectively identifies clinically relevant anatomical changes in patients with LA-NSCLC. Implementation of the protocol was associated with improved target coverage without increasing OAR doses. With appropriate training, integration into routine clinical workflow appears feasible and enables reliable anatomical adaptation. Clinical outcomes appear to be broadly consistent with international data.

## Supplementary Material



## Data Availability

The data presented in this study are available on request from the corresponding author. The data are not publicly available due to privacy reasons as they are part of an ongoing study.
