# Supplementary material for: Prospective evaluation and clinical outcomes of adaptive radiotherapy for locally advanced non-small cell lung cancer (LA-NSCLC)
Source: Acta Oncol. 2026 May 27;65:45745. doi: 10.2340/1651-226X.2026.45745 (PMC13221713; doi:10.2340/1651-226X.2026.45745)
Supplement: Supplementary file 1 [file AO-65-45745-s1.pdf]

Supplementary material has been published as submitted. It has not been copyedited, or typeset by Acta Oncologica

Supplementary material

Traffic Light Protocol for Lung Cancer Patients

Table S1: Traffic light protocol with color coded action levels.

Color code	Description	Action
	Major changes, treatment cannot be delivered on the current plan	Call medical physicist
	Anatomical changes, dose coverage must be evaluated	Initiate control CBCT task (treatment is given this day)
	Some changes, dose coverage assumed acceptable	Note the type of change
	Small or no changes	Mark with green

Atelectasis: Atelectasis may change during radiotherapy and often occurs in relation to the tumor. The extent of atelectasis cannot predict whether replanning is needed, but 70% of all atelectasis require replanning due to both tumor shift and dosimetric changes. Atelectasis in the tumor region should generally be coded orange.

Pleural effusion: Changes in pleural effusion did not alter tumor position, but pleural effusion may affect dose if the beam enters through it. However, changes < 2 cm have little impact on dose distribution. Pleural effusion < 0.5 cm should be coded green, 0.5-2 cm yellow, and >2 cm orange.

Infiltrative changes: Diffuse density changes do not necessarily occur in relation to the tumor, and diffuse changes alone do not indicate replanning. If the density changes do not affect tumor position, they should be coded yellow. If you suspect change in tumor mass and it lies within the PTV, it should be coded orange. If you suspect added tumor mass outside the PTV, it should be coded red.

Baseline shift: Systematic shift in the tumor position relative to bone match requires replanning. Matching on tumor to improve tumor coverage leads to less control of OAR doses and requires extra attention to the spinal cord dose. Baseline shift <2 mm should be coded green, 2-5 mm yellow (maintain bone match), and >5 mm orange (correction to tumor match

can be done but be aware of nodal CTV and spinal cord dose). Baseline shift preventing coverage of both tumor and lymph nodes should be coded red.

<u>Tumor growth:</u> Tumor within PTV while also covering lymph nodes should be coded yellow. Tumor at the PTV boarder should be coded orange. Tumor outside PTV should be coded red.

<u>Tumor shrinkage:</u> In general, the tumor will still be well covered, and considering potential subclinical disease, it is not given that the CTV should be reduced. However, loss of solid tumor mass in the lung may create potential hotspots in normal tissue. Shrinkage of 1-3 cm should be coded yellow and shrinkage of >3 cm orange.

Radiation-related toxicity

Table S2: Radiation-related toxicity according to CTCAE v5.0 in all 46 patients receiving treatment.

Radiation-related toxicity, n = 46	During treatment	3 months	6 months	3 years
Esophagitis, n (%)				
None or grade 1	23 (50.0%	43 (93.5%)	46 (100%)	46 (100%)
Grade 2	16 (34.8%)	3 (6.5%)	0	0
Grade 3	7 (15.2%)	0	0	0
Pneumonitis, n (%)				
None or grade 1	45 (97.8%)	37 (80.4%)	37 (80.4%)	46 (100%)
Grade 2	1 (2.2%)	7 (15.2%)	6 (13.0%)	0
Grade 3	0	2 (4.3%)	3 (6.5%)	0
